# Beyond Ribosomal Mutations: Identification of *MPN_080* as a Novel ATPase-Dependent Determinant of Macrolide Resistance in *Mycoplasma pneumoniae*

**DOI:** 10.3390/microorganisms14040831

**Published:** 2026-04-05

**Authors:** Shaoli Li, Yuyan Xia, Fei Zhao, Xiuwei Wang, Zhengli Li, Liyong Liu, Junting Liu, Mei Diao

**Affiliations:** 1 Capital Institute of Pediatrics-Peking University Teaching Hospital, Beijing 100020, China; 18500302278@163.com; 2 Capital Center for Children’s Health, Capital Medical University, Beijing 100020, China; 3 Capital Center for Children’s Health, Capital Institute of Pediatrics, Capital Medical University, Beijing 100020, China; xyysher@gmail.com (Y.X.); wangxiuwei1010@126.com (X.W.); jocelynbj@126.com (Z.L.); juntingliu@mail.ccmu.edu.cn (J.L.); 4 National Institute for Communicable Disease Control and Prevention, Chinese Center for Disease Control and Prevention, Beijing 102206, China; zhaofei@icdc.cn (F.Z.); liuliyong@icdc.cn (L.L.)

**Keywords:** *Mycoplasma pneumoniae*, erythromycin resistance, *MPN_080*, ABC transporter

## Abstract

*Mycoplasma pneumoniae* is a significant pathogen responsible for community-acquired respiratory infections in children and adolescents, with the rising prevalence of macrolide-resistant *M. pneumoniae* (MRMP), particularly in Asia, presenting critical treatment challenges. Our previous study inferred that a macrolide efflux pump may contribute to macrolide resistance in *M. pneumoniae* in addition to the common point mutations in 23S rRNA gene. This study aimed to define the specific pump and confirm its role. Through comparative genomic analysis, we identified a candidate gene, *MPN_080*, encoding an ABC transporter permease, which was further characterized using phylogenetic analysis, AlphaFold-based structural modeling, and biochemical assays. Overexpression of MPN_080 from an erythromycin-resistant isolate in the erythromycin-sensitive M129 resulted in a significant increase in minimum inhibitory concentrations (MICs) from <0.125 µg/mL to 1 µg/mL, while similar overexpression of MPN_080 derived from M129 did not affect MICs. Notably, this resistance mechanism operates independently of *M. pneumoniae* virulence factors, as evidenced by unaltered colonization capacity in NCI-H292 cells and consistent immune response patterns across both strains. Our findings establish *MPN_080* as a novel determinant of macrolide resistance functioning associated with enhanced ATPase activity. These insights into non-classical resistance mechanisms may guide future diagnostic and therapeutic strategies against MRMP.

## 1. Introduction

*Mycoplasma pneumoniae* (*M. pneumoniae*) is a major cause of community-acquired respiratory infections, particularly affecting children and young adults [1]. Due to its lack of a cell wall, *M. pneumoniae* is inherently resistant to a range of antibiotics that target cell wall synthesis. Although macrolides have traditionally served as the primary treatment option owing to their favorable tissue penetration and safety profile, macrolide-resistant *M. pneumoniae* (MRMP) has become increasingly prevalent worldwide. This trend poses a substantial challenge to clinical management [2]. Recent meta-analyses encompassing 27,408 clinical samples from 26 countries have identified concerning epidemiological trends, with resistance rates surpassing 50% in the Western Pacific region, thereby presenting a significant public health challenge [3].

The principal mechanism underlying macrolide resistance is target-site alterations in the 23S rRNA, notably the A2063G mutation, which is responsible for over 95% of *M. pneumoniae* resistant strains by diminishing drug binding affinity [4]. Additional mechanisms encompass mutations in ribosomal proteins L4 and L22, and, less frequently, the presence of efflux pumps or drug-modifying enzymes [5,6]. Although mutations in the 2063 site constitute the primary mechanism of macrolide resistance in *M. pneumoniae*, emerging clinical data suggest that other mechanisms may exist, particularly in pediatric populations [7].

In our prior study, we performed genomic analysis of ten erythromycin-resistant *M. pneumoniae* isolates [6]. Notably, we identified recurrent mutations in macrolide-specific efflux transporter, and efflux pump inhibitors caused a significant decrease of minimum inhibitory concentrations (MICs), even restoring susceptibility in some strains. These findings suggest that efflux pump mechanism contributes to macrolide resistance in *M. pneumoniae*, alongside the well-documented point mutations in the 23S rRNA gene. In the present study, we focus on the role of a specific gene *MPN_080*, which encodes a protein of the macrolide-specific efflux pump protein of the ATP-binding cassette (ABC) transporter family in *M. pneumoniae* and shares some sequence similarity with the *MacB* efflux pump gene in *Escherichia coli* [8]. We aimed to characterize the genetic variations in *MPN_080* and confirm their contribution to macrolide resistance phenotypes. This study is expected to yield critical insights into the mechanisms underlying treatment failure and inform strategies for combating resistant *M. pneumoniae* infections.

## 2. Materials and Methods

### 2.1. Bacterial Strains and Culture Conditions

The *M. pneumoniae* strain M129 (ATCC 29342) and macrolide-resistant clinical isolates (RC267) were cultivated in mycoplasma broth medium (HB7025-2, Haibo, Qingdao Hope Bio-Technology Co., Ltd., Qingdao, China), which was supplemented with 40% fetal bovine serum at 37 °C. Antibiotic selection for plasmid transformed *M. pneumoniae* was performed using gentamicin at a concentration of 50 µg/mL. The bacterial cultures were monitored for changes in medium color, indicative of the logarithmic growth phase. *E. coli* TransT1 and BL21-CodonPlus(DE3)-RIL were used to clone and express the *MPN_080* gene and were cultured in Luria–Bertani (LB) broth with antibiotics kanamycin (50 µg/mL) or gentamicin (100 µg/mL), respectively. The bacterial strains and plasmids utilized are listed and described in Table S1.

### 2.2. Phylogenetic Analysis

To investigate the evolutionary relationship of MPN_080 protein among different *M. pneumoniae* strains and its homologs, phylogenetic analysis was performed following standardized protocols [9]. First, MPN_080 protein sequences from 14 *M. pneumoniae* strains (including the macrolide-resistant RC267, macrolide-sensitive M129, and 12 additional clinical isolates) were retrieved from the NCBI GenBank database. Homologous sequences of MPN_080 (ATPase proteins from related bacterial species) were also obtained via BLASTp search with M129 MPN_080 as the query sequence, and sequences with a similarity ≥70% and coverage ≥80% were selected for further analysis (accession numbers: WP_159202071.1, WP_058158331.1, WP_3MPN_08010166.1, WP_350260418.1, WP_017532940.1, WP_014325326.1, WP_019830476.1, WP_086136151.1, WP_054173838.1, WP_054175501.1, WP_054175921.1). All retrieved protein sequences were aligned using the MUSCLE algorithm implemented in MEGA11 software (v11.0.13) with default parameters: gap opening penalty of 29, gap extension penalty of 0.2 for the first pass, and gap extension penalty of 0.1 for subsequent passes. The alignment quality was inspected and manually adjusted to eliminate ambiguous regions (e.g., regions with excessive gaps or low sequence conservation) to ensure the reliability of phylogenetic inference. A neighbor-joining (NJ) phylogenetic tree was constructed based on the multiple sequence alignment using MEGA11 [10].

### 2.3. Expression and Purification of the M. pneumoniae MPN_080 Protein

A codon-optimized *MPN_080* gene from *M. pneumoniae* strain RC267 and M129 were synthesized by Sangon Biotech (Shanghai, China) and cloned into the pET-32a(+) vector. During synthesis, the eight tryptophan-encoding UGA codons naturally present in the gene were replaced with TGG to enable correct expression in *E. coli*. The resulting construct encodes a 1385-amino acid recombinant protein fused with a C-terminal His tag. The recombinant plasmid was then transformed into *E. coli* BL21-CodonPlus(DE3)-RIL competent cells. Protein expression was induced with 1 mM isopropyl β-D-1-thiogalactopyranoside (IPTG, Invitrogen), then the expressed protein was purified using a nickel-nitrilotriacetic acid (Ni-NTA) column according to the manufacturer’s instructions (Thermo Fisher Scientific, Waltham, MA, USA).

### 2.4. Vector Construction and Bacterial Transformation

The Strep-Tag II fragment was synthesized and amplified using PCR with specific primers (strepIIF:5′CACACACTAGTACGGATCCAGCCCATGGTGGAGC3′; strepIIR: 5′TCCTCTAGAAAAAGGCCATCCGTCAGGATGGCCTTCTTCTTTATTTTTCGAACTG3′). The purified PCR products were then fused to the linearized pMT85 vector, which had been digested with XbaI and BamHI at 37 °C for 1 h, using the Gibson Assembly cloning Kit (New England Biolabs, Ipswich, MA, USA). The assembled plasmids were introduced into Trans1 competent cells via heat shock. After transformation, the cells were plated on LB agar plates containing 100 µg/mL gentamicin and incubated at 37 °C for 12–16 h. Colonies were screened by colony PCR and sequenced to confirm the successful construction of the pMT85O vector. For the subsequent constructs, pMT85S (sensitive) and pMT85R (resistance), the *MPN_080* gene fragment was amplified from the M129 and RC267 genomic DNA using specific primers. The PCR products were then assembled into the linearized pMT85O vector backbone using the same Gibson Assembly method. Notably, these constructs incorporate the native promoter of the *MPN_080* gene, which consists of a 235 bp upstream region immediately preceding the *MPN_080* coding sequence. This region was included to ensure that the expression of the *MPN_080* gene is regulated by its own promoter, maintaining the natural transcriptional control mechanisms in *M. pneumoniae*.

For the electrotransformation of *M. pneumoniae* M129, cultures in the late-logarithmic growth phase were collected and resuspended in Hepes-sucrose buffer to achieve a final concentration of 10^9^–10^10^ CCU/mL. A 200 μL aliquot of competent cells was combined with 100 μg of either the pMT85O or pMT85R or pMT85S plasmids [11]. The mixture was incubated on ice for 5 min, followed by electroporation at 1.8 kV, 200 Ω, and 25 μF using a Bio-Rad Gene Pulser™ electroporator (Bio-Rad Laboratories, Hercules, CA, USA). Subsequent to electroporation, the cells were suspended in 1 mL of pre-warmed medium and incubated at 37 °C for 1 h to facilitate the expression of the antibiotic resistance marker. Transformants were selected on agar plates containing 50 μg/mL gentamicin. Individual colonies were subsequently transferred into mycoplasma broth medium supplemented with 40% fetal bovine serum and 50 μg/mL gentamicin. Positive clones were confirmed through PCR amplification (*GNATF*: GATACCAGTTCATTTGGGTT,*GNATR*::TGATCCATACCATAGACTATCTC) and Western blot.

### 2.5. Western Blot Analysis

Protein extracts were prepared from positive clones using RIPA lysis buffer supplemented with PMSF. Protein samples (10 µg per lane) were separated by 10% SDS-PAGE and transferred onto a PVDF membrane. The membrane was blocked with 5% skimmed milk in TBST and then incubated overnight at 4 °C with an anti-Strep-tag II primary antibody (1:500 dilution, EnoGene E12-004-1). Following washing, the membrane was incubated with an HRP-conjugated goat anti-mouse secondary antibody (1:50,000 dilution, Boster BA1051) at 37 °C for 2 h. Protein bands were visualized using an ECL substrate and imaged on a ChemiDoc system.

### 2.6. Minimum Inhibitory Concentration (MIC) Assays

Minimum inhibitory concentration (MIC) determinations were conducted in triplicate utilizing 96-well microtiter plates based on the Clinical and Laboratory Standards Institute (CLSI document M43A) completely [12]. *M. pneumoniae* isolates, specifically wild-type M129 and M129-MPN_080 (overexpressing MPN_080), were cultured in mycoplasma broth medium supplemented with 40% fetal bovine serum at 37 °C until the medium exhibited visible yellowing, indicative of logarithmic growth. Serial dilutions of antibiotics were made with final concentration of 128 μg/mL to 0.125 μg/mL; *M. pneumoniae* suspensions were prepared in mycoplasma broth medium containing 10^5^ CFU/mL. Negative controls comprised the strain inoculum without erythromycin. The plates were incubated at 37 °C for a duration of 14 days, until the negative control exhibited a complete color change of the medium. MIC values were determined as the lowest concentration of the drug that completely inhibited visible growth, as evidenced by the absence of color change.

### 2.7. Infection of NCI-H292 Cells with MPN_080 Overexpression Mutants

NCI-H292 cells in the logarithmic growth phase were seeded at a density of 5 × 10^5^ cells per well in a 6-well plate and incubated overnight at 37 °C with 5% CO_2_. Mycoplasma strains were inoculated at a concentration of 10% (*v*/*v*) into 10 mL of mycoplasma broth supplemented with 10% bovine serum and cultured for approximately two weeks until a color change was observed. The culture was subsequently subjected to two-fold serial dilution to determine the color-changing unit (CCU) and adjusted to a concentration of 10^8^ CCU/mL. The prepared mycoplasma suspension was introduced at a multiplicity of infection (MOI) of 50:1, and the cells were incubated at 37 °C with 5% CO_2_ for 18 h. After incubation, the medium was removed, and the cells were washed three times with phosphate-buffered saline (PBS) to remove unbound mycoplasma prior to performing Real-Time Quantitative PCR or transmission Electron Microscopy (TEM).

NCI-H292 cells underwent an initial fixation process in 2.5% glutaraldehyde dissolved in a 0.1 M phosphate buffer (pH 7.4) at 4 °C for a duration of 2 to 4 h, followed by three washes with PBS. Subsequently, secondary fixation was conducted using 1% osmium tetroxide at ambient temperature for 2 h, with subsequent washing. The dehydration process involved a graded ethanol series, and the samples were then infiltrated overnight with a mixture of acetone and 812 resin, followed by infiltration with pure resin overnight. The embedding was carried out at 60 °C for 48 h. Ultrathin sections, measuring 60–80 nm, were stained with 2% uranyl acetate and lead citrate, and allowed to dry overnight prior to transmission electron microscopy (TEM) observation.

### 2.8. PCR

DNA was isolated from infected NCI-H292 cells using the QIAamp Mini DNA kit (Qiagen, Hilden, Germany) according to the manufacturer’s instructions. Bacterial cells number was quantified by real-time PCR performed with the *M. pneumoniae* MpP1 assay [13].

### 2.9. Real-Time Quantitative PCR

Host gene expression analysis was analyzed by quantitative PCRs. Total RNA was isolated from cultured cells utilizing the Trizol method, followed by phase separation with chloroform and precipitation with isopropanol to obtain RNA. The purified RNA was quantified using a NanoDrop 2000 spectrophotometer(Thermo Fisher Scientific, Waltham, MA, USA), and its purity was evaluated based on the OD260/OD280 ratio. Reverse transcription into complementary DNA (cDNA) was conducted using HiScript III RT SuperMix, with genomic DNA being removed prior to the reverse transcription process. For quantitative PCR, a reaction volume of 10 µL per well was prepared in a 384-well plate, comprising 4 µL of cDNA, 1 µL of forward primer (10 µM), 1 µL of reverse primer (10 µM), 22.5 µL of SYBR Green Master Mix, and deionized water to reach a final volume of 45 µL before distribution into the reaction plate. The qPCR cycling conditions were set at 50 °C for 2 min, 95 °C for 5 min, followed by 40 cycles of 95 °C for 15 s, 56 °C for 20 s, and 72 °C for 40 s. The primer sequences used are listed in Table 1.

### 2.10. ATPase Activity Assay

The ATPase activity assay was performed using the EnzChek Phosphate Assay Kit (Cat. No. E6646, Thermo Fisher Scientific, Waltham, MA, USA) to quantify inorganic phosphate release according to the manufacturer’s instructions [13]. Activity was calculated as μmol Pi·μg^−1^ protein based on triplicate measurements.

### 2.11. Statistical Analysis

The experimental data were subjected to statistical analysis utilizing GraphPad Prism v8.0. The results are expressed as the mean ± standard deviation (mean ± SD). Comparative analysis between two groups was conducted using a two-tailed unpaired Student’s *t*-test. For comparisons across multiple groups, a one-way analysis of variance (ANOVA) was employed, followed by Tukey’s post hoc test for multiple comparisons. A *p*-value of less than 0.05 was considered indicative of statistical significance. All experiments were performed in at least three independent biological replicates, with each sample measured in technical triplicate.

## 3. Results

### 3.1. Phylogenetic Analysis and Protein Structure Comparison of RC267 and M129 Strain MPN_080 Proteins

**Figure 1:**
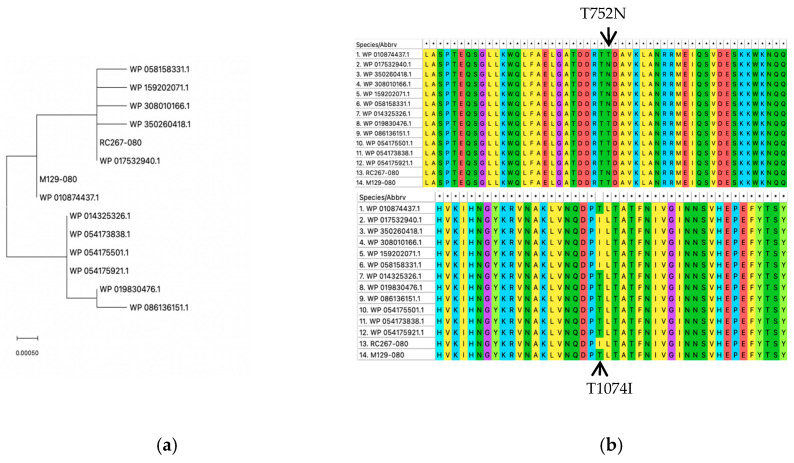
Phylogenetic Analysis and Structural Comparison of the MPN_080 in *Mycoplasma pneumoniae* Strains RC267 and M129. (**a**) Multiple sequence alignment of MPN_080 protein sequences from 14 different isolates using MEGA11, with a neighbor-joining tree generated from the alignment. (**b**) Multiple sequence alignment of MPN_080 with its closest homologous ATPase proteins. Protein sequences included WP_159202071.1, WP_058158331.1, WP_350260418.1, WP_017532940.1, M129-MPN_080, WP_014325326.1, WP_019830476.1, WP_086136151.1, WP_054173838.1, WP_054175501.1, and WP_054175921.1, RC267-080, M129-80. *: the amino acid sequences of 14 species/abbrv at this site are the same.

To assess the evolutionary relationship of MPN_080 protein from the macrolide-resistant strain (RC267), macrolide-sensitive strain (M129) and other clinical isolates, we compared MPN_080 sequences with other *M. pneumoniae* sequences from NCBI database, and constructed a phylogenetic tree using MEGA11. The results demonstrated a high degree of protein sequence similarity between RC267 and M129 (Figure 1a).

To investigate potential functional implications of the *MPN_080* gene, we aligned the amino acid sequences of the MPN_080 protein and its homologs using the MUSCLE algorithm in MEGA11. The data showed that MPN_080 is highly conserved across different M. pneumoniae strains except two amino acid substitutions—T752N and T1074I—identified in resistance strains RC267 (Figure 1b), reflecting consistent evolutionary trends.

### 3.2. Structural Modeling of MPN_080 from RC267 and M129 and Comparison of Putative Interactions Within the ATP-Binding Pocket

To further evaluate the structural and functional consequences of these mutations, we employed AlphaFold to predict and compare the three-dimensional structures of the MPN_080 protein in RC267 (resistant strain) and M129 (susceptible strain).

**Figure 2:**
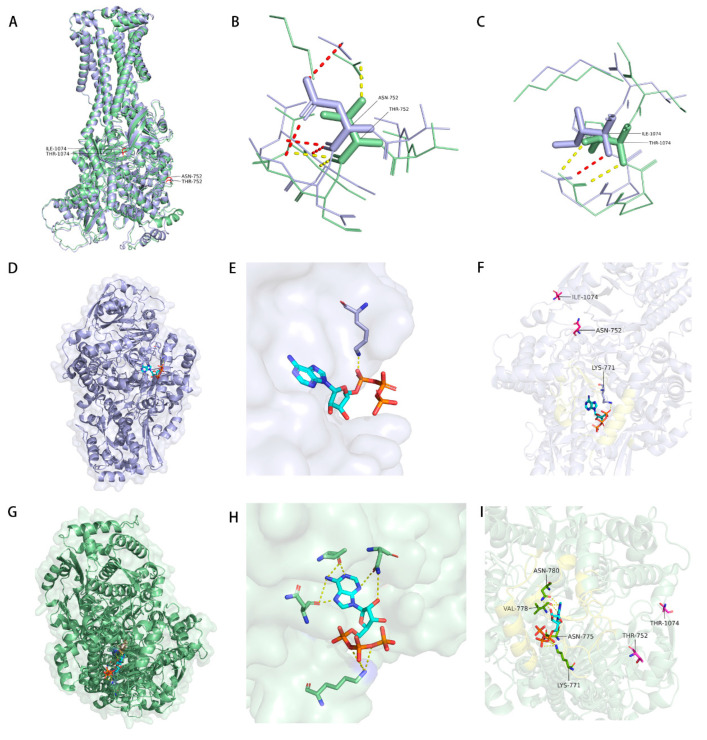
Structural superposition of RC267 and M129 (N752T/I1074T) and comparison of putative interactions within the ATP-binding pocket (Wild type: purple; Mutant type, green). (**A**) Global alignment in PyMOL3.1 using CEalign (default parameters) shows highly similar overall folds (whole-protein RMSD = 1.79 Å). Local deviations near the mutation sites are modest (≈1.57 Å at site 752 and ≈1.65 Å at site 1074), consistent with preserved global architecture and primarily local rearrangements. (**B**) Close-up of site 752 shows a denser set of polar contacts/hydrogen bonds around Asn752 in RC267, whereas Thr752 in M129 supports fewer such interactions, consistent with differences in side-chain chemistry. (**C**) Close-up of site 1074 shows that Ile1074 in RC267, while not providing side-chain hydrogen-bonding capability, offers increased hydrophobic bulk and space filling, with tighter packing around the ATP adenine-ring region. (**D**–**F**) The wild-type ATP complex predicted by AlphaFold3 and rendered in PyMOL places ATP in the expected pocket. Local stabilization is dominated by a putative electrostatic/hydrogen-bond interaction between Lys771 and the ATP γ-phosphate, while the adenine-ring region shows limited additional stabilizing contacts; sites 752/1074 are spatially proximal to the binding interface. (**G**–**I**) In the mutant ATP complex, ATP adopts a similar overall placement, but local contacts are reorganized. Using the same pocket view and hydrogen-bond criteria as in the wild type, residues such as Asn775 and Asn780 form a more extended hydrogen-bonding network, and Val778 shows closer nonpolar contacts (or backbone-mediated interactions) with the ligand.

The overall folds of the mutant (RC267) and wild-type (M129) MPN_080 protein were found to be highly similar by structural alignment in PyMOL 3.1(Schrödinger, LLC, New York, NY, USA) with a RMSD of only 1.79 Å for the whole protein (Figure 2A). The local structural stability was also maintained at the mutation sites with local RMSD values of 1.57 Å and 1.65 Å for T752N and T1074I sites, respectively. The changes in the mutations did not affect the overall architecture of the protein, however, it likely changed the local side-chain physicochemical properties like hydrogen-bonding potential or hydrophobicity (Figure 2B,C).

Using AlphaFold3 to predict the ATP-bound complex unveiled that both variants adopt similar binding conformations; both variants dock ATP at the same pocket without any major topological rearrangement (Figure 2D,G). However, the highly detailed structural comparison suggested local alterations of the ATP-bound physicochemical environment and interaction network (Figure 2E,F,H,I). At position 752, the amide side chain of the mutant Asn752 may have stronger polar interactions and thus be able to form a denser hydrogen-bonding around ATP than the less extensive contacts offered by Thr752 in the wild type. At position 1074, the hydrophobic side chain of Ile1074 was increased which appeared to enhance shape complementarity and packing against the adenine ring of ATP; this presumably reduces the volume of the cavity and improves hydrophobic adhesion. In contrast, in the wild type, the smaller and polar Thr1074 gave less-favorable hydrophobic contact and local filling.

Overall, these structural features suggest that the mutant protein may exhibit enhanced polar contacts around ATP and optimized hydrophobic complementarity near the adenine ring, while the overall shape of the binding pocket remains unchanged. Based on these computational predictions, we hypothesize that the RC267 version of MPN_080 could have stronger binding affinity for ATP, potentially leading to increased ATP hydrolysis and enhanced drug efflux ability, which may contribute to the macrolide-resistant phenotype.

### 3.3. MPN_080 Exhibits ATPase Activity Following Refolding from Inclusion Bodies

**Figure 3:**
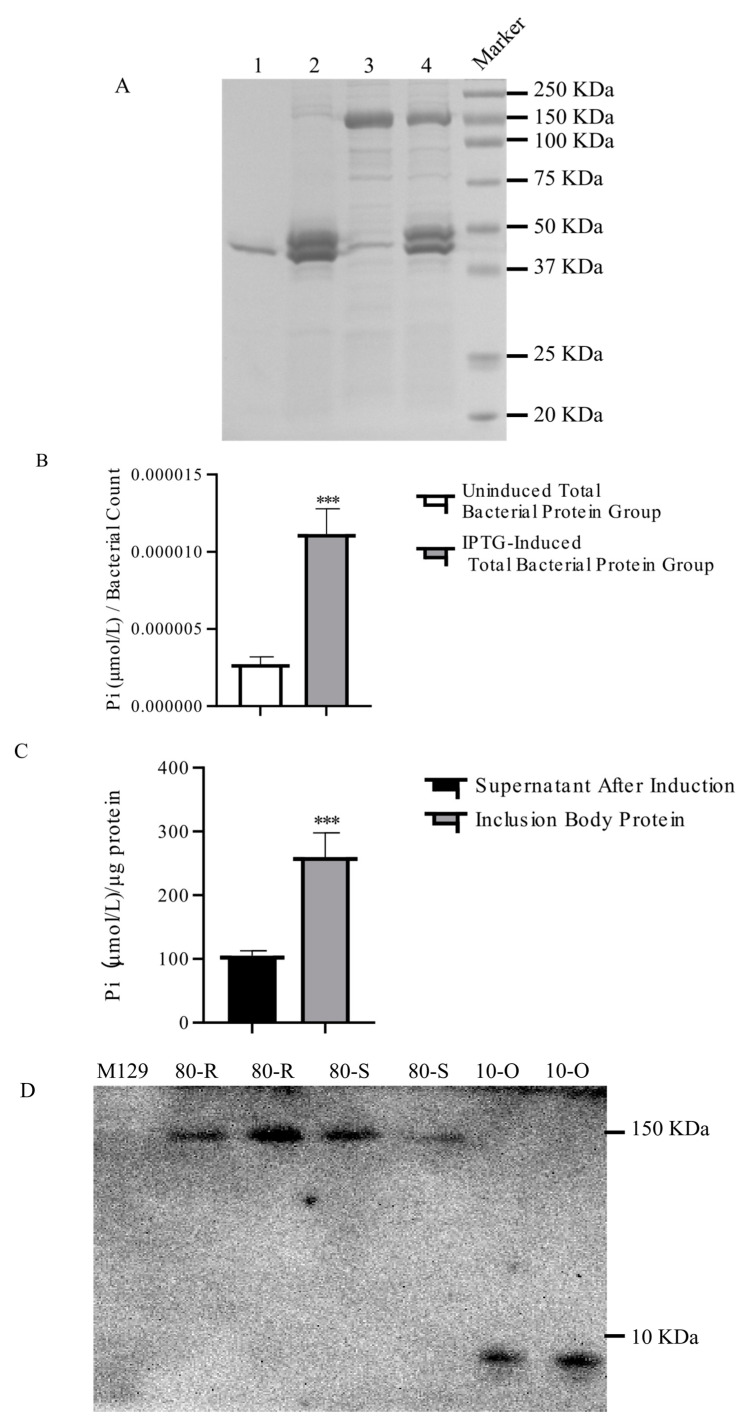
Analysis of MPN_080 Protein Expression in *E. coli* and *M. pneumoniae*. (**A**) SDS-PAGE analysis showing the expression pattern of MPN_080 under different induction conditions. Line 1~4: The 1st to 4th denaturation and refolding product of inclusion bodies precipitation. (**B**) ATPase activity assay of total cellular proteins from *E. coli* expressing MPN_080, with or without IPTG induction (*** *p* < 0.001). (**C**) Comparison of ATPase activity between purified soluble (supernatant) and MPN_080 protein refolded from inclusion bodies (*** *p* < 0.001). (**D**) Western blot confirming expression of Strep-tagged MPN_080 in transformed *M. pneumoniae* M129 strains. 80-O: M129 transformed with empty vector control (pMT85O, contains Strep-tag II only). 80-S: M129 transformed with pMT85S overexpressing wild-type *MPN_080* from strain M129. 80-P: M129 transformed with pMT85R overexpressing mutant *MPN_080* from strain RC267. The anti-Strep-tag II antibody detects the tagged MPN_080 protein (~150 kDa).

To investigate the potential function of the MPN_080 protein in the RC267 strain, we cloned the corresponding gene into the pET-32a(+) expression vector and induced its expression in *E. coli* BL21-CodonPlus(DE3)-RIL cells. SDS-PAGE analysis revealed that the recombinant protein was primarily expressed in the form of inclusion bodies (Figure 3A). Next, we assessed the ATPase activity of MPN_080 by measuring the release of inorganic phosphate (Pi) during ATP hydrolysis. The results showed that the total protein extracted from induced cells exhibited significantly higher ATPase activity than that from uninduced controls (Figure 3B), suggesting that MPN_080 may possess ATP hydrolyzing capability.

Furthermore, we evaluated the ATPase activity of both soluble protein and refolded inclusion body protein (Figure 3C). Remarkably, the refolded protein retained substantial ATPase activity. This indicates that correct folding is likely critical for its enzymatic function and supports the hypothesis that MPN_080 functions as an active ATPase.

### 3.4. RC267-Derived MPN_080 Confers Increased Erythromycin MIC in M129

To identify if MPN_080 plays a functional role in conferring erythromycin resistance in *M. pneumoniae*, MIC assays were conducted. Two recombinant plasmids, pMT85R (introduced MPN_080 from erythromycin-resistant strain RC267) and pMT85S (introduced MPN_080 from erythromycin-sensitive strain M129), were designed and transferred separately into M129 to yield overexpression transformants M129R and M129S, respectively. Western Blot revealed that exogenous *MPN_080* genes from both sources delivered by the recombinant plasmids were expressed (Figure 3D). PCR analysis revealed that the exogenous *MPN_080* genes were chromosomally integrated into the parent M129 genome.

Wild-type M129 exhibited an MIC value of <0.125 μg/mL against erythromycin, while the MIC of the MPN_080 overexpression transformant M129R increased to 1 μg/mL. In contrast, M129S transformant overexpressing WT MPN_080 did not possess this resistance phenotype (MIC < 0.125 μg/mL, Table 2).

### 3.5. MPN_080 Does Not Affect the Colonization Ability of M. pneumoniae in NCI-H292 Cells

**Figure 4:**
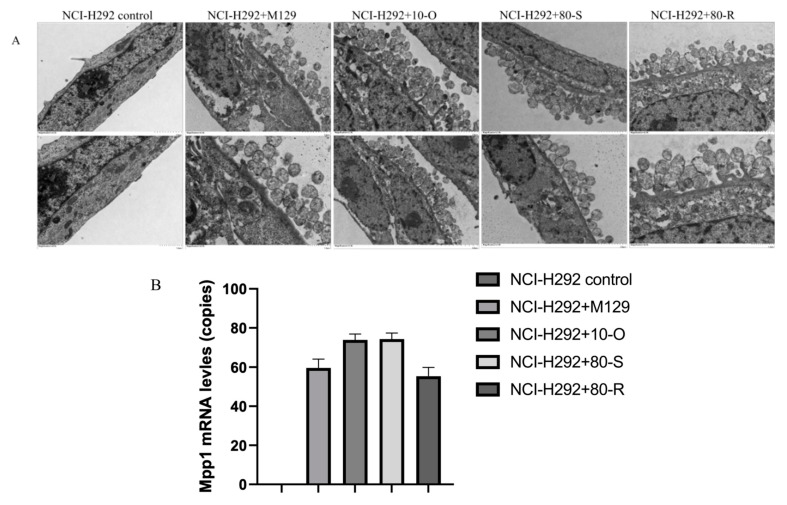
MPN_080 Does Not Affect the Colonization of *M. pneumoniae* in NCI-H292 Cells. (**A**) Transmission electron microscopy (TEM) images showing colonizations of *M. pneumoniae* in NCI-H292 cells. Scale bar: 500 nm. (**B**) Quantification of bacterial load by quantitative PCR targeting the *MpP1* gene, which encodes an adhesion-related protein.

To assess the potential influence of MPN_080 on the colonization ability of *M. pneumoniae*, we conducted an in vitro infection assay using the human lung epithelial cell line NCI-H292. TEM analysis revealed substantial accumulation of *M. pneumoniae* M129 parent or transformants on the surface of infected cells, indicating efficient adherence (Figure 4A). Real-time PCR analysis of *MpP1*, a gene encoding an adhesion-related protein in *M. pneumoniae*, was used to quantify the number of bacteria in the infected groups. The analysis revealed no significant differences among cells infected with parent, WT or mutant MPN_080 overexpression tranformants (Figure 4B). These data suggest that overproduction of both WT and mutant MPN_080 from RC267 and M129 does not affect the fitness of the bacteria colonization.

### 3.6. MPN_080 Does Not Affect the Immune Responses of M. pneumoniae

**Figure 5:**
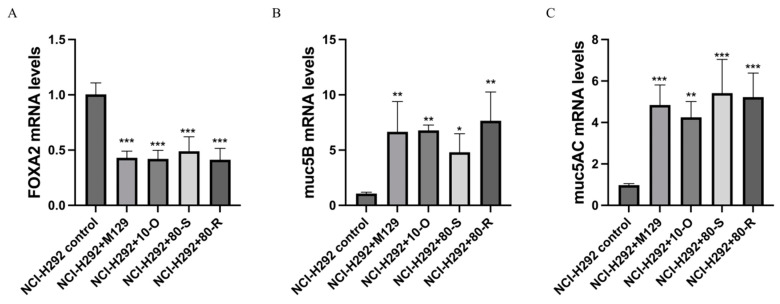
Expression Levels of *muc5AC*, *muc5B*, and *FoxA2* mRNA in NCI-H292 Cells Infected with Different *M. pneumoniae* Strains (* *p* < 0.05, ** *p* < 0.01, *** *p* < 0.001 versus the control group). (**A**) Quantitative real-time PCR (qRT-PCR) was used to analyze the mRNA expression of *FoxA2*, in cells infected with the wild-type M129, M129O, M129S, and M129R. (**B**) qRT-PCR was used to analyze the mRNA expression of *muc5B*,  (**C**) qRT-PCR was used to analyze the mRNA expression of *muc5AC*.

In order to investigate the possible impact of MPN_080 on host immune response, we examined the expression of *muc5AC*, *muc5B* and transcription factor *foxA2* in NCI-H292 Cells infected with different strains. Results of qRT-PCR (Figure 5) showed highly significantly upregulated expression of *muc5AC* and *muc5B* and significantly downregulated expression of *foxA2* (*p* < 0.001) in all infection groups compared to uninfected control. However, no significant differences were evidenced among the various MPN_080 expression backgrounds (wild-type, empty vector control and two overexpression constructs), indicating that host mucosal immune responses are not influenced by MPN_080.

## 4. Discussion

*M. pneumoniae* is a significant etiological agent of community-acquired pneumonia, particularly among children and young adults, and contributes to a substantial burden of respiratory morbidity worldwide [14]. Due to its high prevalence among pediatric populations and the limited use of alternative antibiotics in children, macrolide antibiotics have been used as the primary first-line therapeutic agents. Despite the relatively recent adoption of this treatment strategy, the resistance rate to macrolides has escalated rapidly [15,16,17,18,19]. Notably, during the global pandemic of 2023–2024, resistance rates in certain regions of China have surged to as high as 99% [20]. This poses a substantial public health challenge, prompting critical inquiries about whether novel mechanisms contribute to drug resistance, and whether these mechanisms can be exploited as therapeutic targets in the future.

Macrolide antibiotics exert their antibacterial effects by binding to the large subunit of the bacterial ribosome, thereby obstructing peptide chain elongation. They also disrupt proper ribosome assembly, ultimately inhibiting protein synthesis [14]. The primary mechanism involves alterations in ribosomal targets, particularly mutations in the 23S rRNA gene—most notably at position A2063—which directly diminish antibiotic binding efficiency [21]. Additionally, mutations in ribosomal proteins L4 and L22 can alter the conformation of the peptide exit tunnel, further impacting drug binding [22]. Beyond these structural mechanisms, some pathogens express erm genes that encode rRNA methyltransferases, which modify rRNA and modify the drug-binding site [23]. Streptococcus pneumoniae employ efflux systems encoded by mefA and mefE, which actively expel macrolides from the cell, thereby reducing their intracellular concentrations and effectiveness [24]. Currently, only point mutations in the 23S rRNA gene and ribosomal proteins L4 and L22 genes have been reported in the resistance mechanism of *M. pneumoniae*.

Our previous research showed that SNPs associated with macrolide-resistant *M. penumoniae* strains were focused on the *MacB* gene, which belongs to macrolide-specific efflux system gene encoding a macrolide ABC transporter ATP-binding protein [6], and the efflux pump inhibitors could decrease the MIC values. These findings are consistent with a model where an active efflux mechanism may contribute to drug resistance, although direct confirmation of efflux activity is needed.

In Gram-negative bacteria, MacB, a novel membrane transporter protein belonging to the bacterial ABC superfamily, together with MacA and TolC, formed a transmembrane complex that harnesses energy from ATP hydrolysis to transport a variety of biologically active molecules and polypeptide virulence factors from the cytoplasm, including macrolide antibiotics [25]. In the case of *M. pneumoniae*, we identified an ABC transporter permease, MPN_080, which shares sequence homology and predicted structural features with the MacB protein a component of the MacAB-TolC efflux pump in *E. coli*. This homology, combined with our functional data, led us to hypothesize that MPN_080 may participate in a similar, yet-to-be-fully characterized efflux mechanism.

Through detailed functional analysis, we discovered two significant mutations (T752N and T1074I) in the ATPase MPN_080 in a strain with high-level erythromycin resistance. Functional tests confirmed that this mutated variant maintains ATPase activity and can increase erythromycin MIC by >8-fold when overexpressed in susceptible strains. Meanwhile, overexpressing WT MPN_080 did not cause any changes in MIC values. These results indicated its role in efflux-mediated resistance mechanism beyond canonical ribosomal pathways. ATPases, as crucial energy-converting enzymes on the cell membrane, are vital for maintaining membrane potential, ion balance, and transmembrane transport. Previous research has shown that changes in other mycoplasma membrane structure can significantly impact antibiotic translocation efficiency [26]. Specifically, increased membrane lipid fluidity may alter membrane permeability, affecting antibiotic accumulation [27]. Additionally, antibiotics like erythromycin and tetracycline not only inhibit protein synthesis but also enhance the membrane lipid fluidity of Chlamydia psittaci, causing conformational and functional changes in the membrane [28].

An intriguing finding of our study is that overexpression of wild-type MPN_080 did not confer erythromycin resistance, whereas the mutant variant (T752N, T1074I) increased MIC by 8-fold. Both substitutions lie outside canonical ATP-binding motifs, arguing against a simple gain-of-function in basal ATP hydrolysis. Structural modeling shows that in the mutant, Asn752 forms a denser polar network around ATP and Ile1074 packs more tightly against the adenine ring. These localized changes do not remodel the binding pocket but could reshape substrate coupling; specifically, they may enable ATP hydrolysis to drive conformational changes that recognize and translocate macrolides, a task the wild-type protein cannot accomplish. This aligns with the observation that only the mutant elevates MIC upon overexpression. Whether the mutations alter substrate specificity, improve hydrolysis-transport coupling, or enable interaction with unknown partners remains open; distinguishing these will require direct transport assays using purified proteins reconstituted in proteoliposomes.

Previous research has demonstrated that mutations in ATP-binding motifs, such as Walker A/B sites, can significantly enhance ATP hydrolysis efficiency, thereby augmenting efflux pump activity [29]. Consequently, the mutations identified in MPN_080 in strain RC267 may enhance its ATPase activity, providing increased energy input to ABC transporters and improving drug efflux efficiency. Beyond drug efflux, surface-associated ATPases like OppA have been implicated in immune evasion and biofilm formation.

It is well-established that MUC5AC and MUC5B are the primary mucin components in respiratory mucus, playing crucial roles in maintaining the airway mucosal barrier and facilitating mucociliary clearance [30]. In pediatric cases of *M. pneumoniae* pneumonia, the pathogen may upregulate the expression of MUC5AC and MUC5B through the activation of the STAT6-STAT3 and epidermal growth factor receptor signaling pathways, leading to excessive mucus secretion and the formation of mucus plugs characteristic of plastic bronchitis [31]. FOXA2, which functions as a transcriptional repressor, can bind to the promoter region of the *MUC5AC* gene and inhibit its transcriptional activity [31]. Following infection with *M. pneumoniae*, there is a significant downregulation of FOXA2 expression, resulting in the overexpression of MUC5AC and contributing to mucus hypersecretion [32]. In this study, despite the implications related to resistance, our colonization and host response assays revealed that the overexpression of MPN_080 did not impact bacterial adhesion to epithelial cells, nor did it significantly influence the expression levels of mucin-related genes (*MUC5AC* and *MUC5B*) or the transcription factor *FOXA2*. The regulation of these host factors remained consistent across all infected groups, regardless of the expression levels of MPN_080.

In conclusion, this study identifies *MPN_080* as a novel ATPase-dependent mediator of macrolide resistance in *M. pneumoniae*. The structural characterization of the transmembrane domains of MPN_080 provides a foundation for the development of targeted efflux inhibitors, while its mutation profile serves as a potential biomarker for clinical resistance screening. Beyond diagnostic applications, a detailed understanding of MPN_080’s substrate specificity may facilitate the rational design of improved macrolide derivatives. Collectively, these findings significantly enhance our understanding of mycoplasma resistance mechanisms and present new opportunities for addressing the escalating challenge of antibiotic resistance.

Nonetheless, certain limitations were encountered in this study. Our efforts to identify interacting partners through pull-down experiments were unsuccessful, possibly due to transient or low affinity interactions, independent functioning of MPN_080 as a transmembrane transporter, or the need for alternative experimental approaches to characterize its protein partners. Furthermore, direct evidence of MPN_080-mediated drug efflux was not obtained; while our structural, biochemical, and overexpression data are consistent with an efflux-related function, they are suggestive rather than conclusive. A direct comparison of ATPase activity between purified wild-type and mutant MPN_080 proteins was not performed in this study; such an experiment would provide stronger evidence for the functional impact of the T752N and T1074I mutations. Our conclusions are also based primarily on a single resistant isolate (RC267) and a single susceptible strain (M129), and validation in a larger panel of clinical isolates is needed to confirm whether the identified mutations represent a generalizable resistance mechanism. Consequently, whether *MPN_080* mutations alone are sufficient to confer clinically relevant resistance, or whether they act primarily in synergy with 23S rRNA mutations, remains to be determined. The modest MIC increase conferred by mutant MPN_080 overexpression (from <0.125 μg/mL to 1 μg/mL) also warrants caution regarding its clinical relevance in isolation, particularly given that the original RC267 isolate harbors both *MPN_080* mutations and the canonical *A2063G* 23S rRNA mutation. Finally, all experiments were conducted in vitro, and the role of *MPN_080* in resistance within the complex in vivo environment requires further investigation.

## Figures and Tables

**Table 1:** Primers used in this study.

Primer Name	Sequence
Homo *GAPDHF*	5′-TCAAGAAGGTGGTGAAGCAGG-3
Homo *GAPDHR*	5′-TCAAAGGTGGAGGAGTGGGT-3
Homo *MUC5ACF*	5′-CGACCTGTGCTGTGTACCAT-3′,
Homo *MUC5ACR*	5′-GTGCAGGGTCACATTCCTCA-3′
Homo *MUC5BF*	5′-AACTGCACCGTGTACCTCTG-3′
Homo *MUC5BR*	5′-TCGTGTTGATGCGGACTTGA-3′
Homo *FOXA2F*	5′-TGTTCGAGAACGGCTGCTAC-3′
Homo *FOXA2R*	5′-GAGTGAGGCGACTCGGTG-3′

**Table 2:** Minimum inhibitory concentrations (MIC) of erythromycin against *Mycoplasma pneumoniae* strains.

Strain	MIC (μg/mL)	Interpretation
*M. pneumoniae M129* (Wild-type M129)	<0.125	Sensitive
*M. pneumoniae M129-080* (RC267)	1	Resistant

## Data Availability

The original contributions presented in this study are included in the article. Further inquiries can be directed to the first author.

## Supplementary Materials

The following supporting information can be downloaded at: https://www.mdpi.com/article/10.3390/microorganisms14040831/s1, Table S1. Strains used in this study.
