# Supplementary material for: Beyond Ribosomal Mutations: Identification of MPN_080 as a Novel ATPase-Dependent Determinant of Macrolide Resistance in Mycoplasma pneumoniae
Source: Microorganisms. 2026 Apr 5;14(4):831. doi: 10.3390/microorganisms14040831 (PMC13118816; doi:10.3390/microorganisms14040831)
Supplement: Supplementary file 1 [file microorganisms-14-00831-s001.zip › microorganisms-4195886-Table S1.pdf]

Table S1. Strains used in this study

Strains	Description and Genotype	Source
E.coli Trans1-T1	Used for plasmid construction	Laboratory stock
E.coli BL21-CodonPlus(DE3)-RIL	Used for protein expression	Laboratory stock
M. pneumoniae M129	Wild-type strain	Laboratory stock
M.pneumoniae RC267	Wild-type strain	Laboratory isolation
M. pneumoniae 10-O	*M. pneumoniae* M129+strepII operon	This study
M. pneumoniae 80-S	*M. pneumoniae* M129+ *M. pneumoniae* M129 $MPN\_080_{strepII}$ operon	This study
M. pneumoniae 80-R	*M. pneumoniae* M129+ *M. pneumoniae* RC267 $MPN\_080_{strepII}$ operon	This study
